# Estimation of colorectal adenoma recurrence with dependent censoring

**DOI:** 10.1186/1471-2288-9-66

**Published:** 2009-09-29

**Authors:** Chiu-Hsieh Hsu, Qi Long, David S Alberts

**Affiliations:** 1 Division of Epidemiology and Biostatistics, College of Public Health, University of Arizona, Tucson, AZ, 85724, USA; 2 Arizona Cancer Center, College of Medicine, University of Arizona, Tucson, AZ, 85724, USA; 3 Department of Biostatistics and Bioinformatics, School of Public Health, Emory University, Atlanta, GA, 48109, USA

## Abstract

**Background:**

Due to early colonoscopy for some participants, interval-censored observations can be introduced into the data of a colorectal polyp prevention trial. The censoring could be dependent of risk of recurrence if the reasons of having early colonoscopy are associated with recurrence. This can complicate estimation of the recurrence rate.

**Methods:**

We propose to use midpoint imputation to convert interval-censored data problems to right censored data problems. To adjust for potential dependent censoring, we use information from auxiliary variables to define risk groups to perform the weighted Kaplan-Meier estimation to the midpoint imputed data. The risk groups are defined using two risk scores derived from two working proportional hazards models with the auxiliary variables as the covariates. One is for the recurrence time and the other is for the censoring time. The method described here is explored by simulation and illustrated with an example from a colorectal polyp prevention trial.

**Results:**

We first show that midpoint imputation under an assumption of independent censoring will produce an unbiased estimate of recurrence rate at the end of the trial, which is often the main interest of a colorectal polyp prevention trial, and then show in simulations that the weighted Kaplan-Meier method using the information from auxiliary variables based on the midpoint imputed data can improve efficiency in a situation with independent censoring and reduce bias in a situation with dependent censoring compared to the conventional methods, while estimating the recurrence rate at the end of the trial.

**Conclusion:**

The research in this paper uses midpoint imputation to handle interval-censored observations and then uses the information from auxiliary variables to adjust for dependent censoring by incorporating them into the weighted Kaplan-Meier estimation. This approach can handle a situation with multiple auxiliary variables by deriving two risk scores from two working PH models. Although the idea of this approach might appear simple, the results do show that the weighted Kaplan-Meier approach can gain efficiency and reduce bias due to dependent censoring.

## Background

Most of the colorectal polyp prevention trials recruit individuals who have undergone removal of a colorectal adenoma within six months prior to study to evaluate a specific preventive agent. Each participant is treated with the preventive agent or the matched placebo for three or more years and then evaluated on occurrence of newly discovered adenomas by performing colonoscopy at follow-up to remove all new colorectal polyps. The follow-up colonoscopy is only scheduled to be performed once at the end of the trial (e.g. at three years after start of the intervention). The actual recurrence time for each participant is then only known as occurring either before or after three years. Based on the nature of the study design, a reasonable statistical method to analyze the polyp data will be logistic regression, which simply analyzes the binary outcomes, i.e. recurrence status at the end of the trial.

Some participants could have their only follow-up colonoscopy before three years or even have more than one follow-up colonoscopy due to health issues. This could introduce potential interval-censored observations, also known as current status data ("case 1" interval-censored data), into the recurrence status data. In addition to interval-censored observations, there could be right censored observations as well if a participant did not have any newly discovered adenomas at all follow-up colonoscopies. Logistic regression could produce a biased estimate of the recurrence rate at the end of the trial in presence of right censored observations before the end of the trial [1]. To account for censoring, modified logistic regression using a weight function, a function of follow-up length, was used to estimate the recurrence rate at the end of the trial [1]. Another potential estimator is the nonparametric maximum likelihood estimator (NPMLE) [2,3], which is often used to analyze interval-censored data. Both the weighted logistic regression and the NPMLE approach assume that censoring is independent of risk of recurrence. Often the reasons (e.g. family history of colorectal cancer and previous polyp history) of a participant having early colonoscopy are associated with risk of recurrence and could, furthermore, induce dependent censoring into the data if some of participants with early colonoscopy are censored before the end of the trial. When the censoring is dependent of risk of recurrence, both the weighted logistic regression and the NPMLE approach could produce a biased estimate of the recurrence rate at the end of the trial.

The variables associated with risk of recurrence can be considered as auxiliary variables and used to recover information for interval-censored observations. There are several methods proposed to analyze interval-censored data with auxiliary variables [4-10]. Of the proposed methods, most of them either focus on discovering the association between the event times and the auxiliary variables [4,6-9] or rely on the assumption of independent censoring [4,6-8,10]. The main interest of this paper is in estimating the marginal recurrence rate at the end of the trial in a situation with multiple auxiliary variables and potential dependent censoring. Only one of the above methods is proposed to estimate the marginal survival function and can adjust for dependent censoring through using the information from auxiliary variables [5]. This method directly uses the estimated censoring distribution conditional on auxiliary variables to adjust for dependent censoring. In a situation when 50% of the observed time points are at the end of the trial, it is possible that reliable estimates of the censoring distribution conditional on multiple auxiliary variables might be difficult to obtain, especially when the sample size is small. Hsu et al. converted interval-censored data problems to right censored data problems using auxiliary variables via multiple imputation in a situation with independent censoring to estimate the marginal survival distribution [10]. To conduct the imputation, they fitted a working failure-time proportional hazards (PH) model to the midpoint imputed event time data to define an imputing risk set for each censored observation. In this paper we adapt and generalize their ideas to handle the case of interval-censored data with potential dependent censoring. We propose to fit two working PH models to derive two risk scores to reduce the auxiliary variables into two scalars that are combinations of the auxiliary variables. One is for the time to recurrence based on the midpoint imputed data and the other is for the time to censoring based on the observed censoring time data. The two working PH models will not be directly incorporated into estimation and are only used to derive two risk scores to summarize the complex structure of auxiliary variables into two scalars. These two risk scores are then used to modify the weighted Kaplan-Meier (WKM) estimation, which is often used to incorporate auxiliary variables into survival analysis to improve estimation [11,12]. In particular, the two risk scores will be categorized to define risk groups to perform the WKM estimation. If the auxiliary variables used to define the risk groups are predictive of recurrence, the analyses using the information from the auxiliary variables should be more efficient than the analyses without using the information. In addition, if the auxiliary variables are also predictive of censoring, the analyses using the information from the auxiliary variables can reduce bias due to dependent censoring. The midpoint imputation approach is attractive because it does not require a distribution for the imputation. Such a distribution would be hard to estimate in a typical colorectal polyp prevention trial because often over 50% of the participants had their only follow-up colonoscopy performed at the end of trial and those participants provided very little information with regard to their actual time of recurrence. It has been shown that midpoint imputation could produce biased survival estimates, especially at early time points [13-15]. The main interest of this paper is in estimating the recurrence rate at the end of the trial, not in estimating the recurrence rate throughout the whole study period. We previously showed that the Kaplan-Meier (KM) estimator based on the midpoint imputation will not produce a biased estimate of recurrence rate at the end of the trial under an assumption of independent censoring [16]. This provides a theoretical foundation for using the midpoint imputed data to replace the interval-censored data and can be generalized to handle a situation that censoring depends on auxiliary variables, when the main interest is in estimating the recurrence rate at the end of the trial. In this paper, we are interested in comparing the performance of the KM and WKM methods derived from the midpoint imputed data with the NPMLE, logistic regression (Logit) and weighted logistic regression (WLogit) methods in both situations of independent and dependent censoring with auxiliary variables.

This paper is organized as follows. In Section 2, we define notation and describe the WKM estimator derived from the midpoint imputed data for interval-censored data with multiple auxiliary variables. In Section 3, we apply the WKM method to a data set from a ursodeoxycholic acid colorectal polyp prevention (UDCA) study. In Section 4, we study properties of the method for finite sample sizes through simulation. A discussion follows in Section 5.

## Methods

Let *X *denote time to first recurrence of adenomas, *T*_*k *_denote the *k*^*th *^follow-up colonoscopy time, where *k *= 1,..., *K*, *τ *(e.g. three years) denote the maximum follow-up time. We only know *X *falls in some interval (*L*, *R*], where *L *<*X *≤ *R*. Right censoring is equivalent to *R *= ∞. Let (*L*_*i*_, *R*_*i*_] denote the observable random interval, (*l*_*i*_, *r*_*i*_] denote the observed time interval and *δ*_*i *_= *I*(*R*_*i *_< ∞) denote the observed recurrence indicator for subject *i*. Suppose there are *n *participants in a study. The observed data are thus *O *= {(*l*_1_, *r*_1_, *δ*_1_),..., (*l*_*n*_, *r*_*n*_, *δ*_*n*_)}. We assume that these *n *subjects come from a random sample and are independent. Each participant could have only one follow-up colonoscopy (i.e. *K *= 1) or more than one follow-up colonoscopy (i.e. *K >*1). For a participant with only one follow-up colonoscopy, the recurrence time is either right censored at *L *= *T*_1 _or interval censored into an interval (0, *R *= *T*_1_]. Of the participants with multiple colonoscopy, some could have their final follow-up colonoscopy at the end of the trial (i.e. *T*_*K *_= *τ*). Therefore, the recurrence time is either right censored at *L *= *T*_*K *_(last follow-up colonoscopy time) or interval censored into an interval (*L*, *R*], where *L *<*X *≤ *R *= *T*_*K *_≤ *τ*.

For participant *i *with an interval-censored recurrence time, i.e. (*l*_*i*_, *r*_*i*_], where *r*_*i *_≤ *τ*, midpoint imputation is used to impute time to recurrence by (*l*_*i *_+ *r*_*i*_)/2, the midpoint of (*l*_*i*_, *r*_*i*_], and time to censoring is either observed at *r*_*i *_when *r*_*j *_<*τ *or right censored at *r*_*j *_when *r*_*j *_= *τ *. For participant *j *with a right censored recurrence time, i.e. *r*_*j *_= ∞, time to recurrence is treated as right censored at *l*_*j*_, where *l*_*j *_≤ *τ *and time to censoring is either observed at *l*_*j *_when *l*_*j *_<*τ *or right censored at *τ *when *l*_*j *_= *τ *. Let X* denote the observed time to recurrence derived from midpoint imputation. That is, X* = *l *if *δ *= 0 and X* = (*l *+ *r*)/2 if *δ *= 1. The KM and WKM estimates can then be derived from the imputed data set.

### WKM estimator with multiple auxiliary variables

Often there are several auxiliary variables in a colorectal polyp prevention trial. These auxiliary variables could be also associated with risk of censoring and incorporated into analysis to reduce bias due to dependent censoring in estimation of the recurrence rate. Let **Z **= {**z**_**1**_,..., **z**_**p**_} denote the *p *auxiliary variables. They could be either categorical or continuous. The WKM method cannot directly incorporate those *p *auxiliary variables into estimation because it requires to categorize those auxiliary variables. One potential solution is to dichotomize each auxiliary variable into two groups and then derive the WKM estimator based on the resulting 2^*p *^categorized groups. With this strategy the number of groups increases with the number of the auxiliary variables, which could be problematic in both consistency and variation of the estimator, especially in a situation with a small sample size. In order to use the information from the auxiliary variables to improve the marginal survival estimate, Hsu et al. considered a situation of right censored data with possibly multiple time-independent or time-dependent continuous covariates and proposed deriving two risk scores [17]. These two risk scores summarize the associations between the covariates and the failure and censoring times from two working PH models, one for the failure time and one for the censoring time. They showed that if one of the two working models is correctly specified, failure time is independent of censoring time conditional on the two risk scores. They also demonstrated in simulations that by incorporating auxiliary variables into survival analysis one can both increase efficiency and reduce bias due to dependent censoring while estimating the marginal survival distribution.

In this paper we adapt and modify the ideas [17] to incorporate multiple auxiliary variables into the WKM method for a situation of interval-censored data with potential dependent censoring. We propose to first fit a working PH model to the midpoint imputed recurrence time to reduce the auxiliary variables to a risk score, which provides an indicator of an individual's risk of recurrence. The risk scores are defined as , where  denote the estimates of the regression coefficients for the working PH recurrence time model. We then fit a working PH model to the observed censoring time to reduce the auxiliary variables to a risk score, which provides an indicator of an individual's risk of censoring. The risk scores are defined as , where  denote the estimates of the regression coefficients for the working PH censoring time model. The two risk scores will be continuous and can be categorized into groups based on dichotomization or quartiles. In a dependent censoring situation, these two risk scores could be highly correlated and thus induce few observations in some of the groups. To overcome this sparseness problem we propose using principal component analysis on the two standardized risk scores (centered and scaled) to derive two orthogonal components (linear combinations of two risk scores) and then categorize these two components separately based on their percentiles into *G*(= I * *J*) groups, where *I *is the number of categories for the first component and *J *is the number of categories for the second component. The WKM estimator can then be easily derived based on the I * *J *categorized groups (denoted as *WKM*^*r *+ *c*^(*I*, *J*)). The hope is that conditional on the two components independent censoring can be induced. To see the magnitude of bias reduction, we will also study the WKM method using either one of the two risk scores to define *G *risk groups (denoted as *WKM*^*r *^(*G*) if only the recurrence time risk score is used and *WKM*^*c*^(*G*) if only the censoring time risk score is used). Note if there is only one auxiliary variable or few categorical auxiliary variables, say two, then there is no need to fit the two working PH models. The auxiliary variable(s) can be directly used to define risk groups.

Assume the categorical risk group variable *G *takes on values 1,..., I * *J*. We illustrate the *WKM*^*r*+*c*^(*I*, *J*) method below. The survival function derived from the imputed data can be written as

where *θ*_*g *_is the probability that a subject is in group *g *and  is the probability of survival for group *g*. Based on the above expression, the WKM estimator^11,12 ^using both of the two risk scores to create the risk groups is defined as , where  is the KM estimator among those in group *g*, *n*_*g *_is the number of subjects in group *g*, and . The recurrence rate at the end of the trial (*τ*) is then equal to 1 - *WKM*^*r*+*c*^(*I*, *J*) (*τ*). The associated variance is equal to the sum of the weighted averages of within-variation and between-variation (see below)

where *λ*(.) and *H*(.) are hazard and cumulative hazard functions, respectively. The first term of the variance can be easily estimated by calculating the weighted average of the variances derived from the Greenwood's formula for those I * *J *groups and the second term can be estimated by plugging in estimates of each component^12^. The same technique can be used to derive *WKM*^*r*^(*G*) and *WKM*^*c*^(*G*).

## Results

### Application to UDCA data

In 1996, the Arizona Cancer Center initiated a multi-center trial to determine whether ursodeoxycholic acid (UDCA) can prevent the recurrence of colorectal adenomas [18]. A total of 1285 subjects identified colorectal adenomas at the qualifying examination were recruited and randomly assigned to one of the two treatment groups, placebo and UDCA (8-10 mg/kg/day). Of 1285 subjects, a total of 1192 subjects underwent at least one follow-up colonoscopy and were thus considered for the endpoint analysis, 579 in the placebo group and 613 in the UDCA group. For each of the 1192 subjects, his/her recurrent status was measured, as well as the baseline covariates, such as age (mean: 66.2; standard deviation: 8.5), gender (67.4% male), BMI (mean: 27.4; standard deviation: 4.6), family history of colorectal cancer (27.4% with family history of colorectal cancer) and previous polyp history (before the qualified examination) (47.3% with previous polyp history). According to the baseline covariates, on average the UDCA participants were slightly overweight and had a higher risk of recurrence compared to the general population.

Initially, the follow-up colonoscopy was planned to be performed only once, at least 30 months after randomization. However, some participants went through their follow-up colonoscopy before the planned time. The number of participants who had early colonoscopy are 233 (40.2%) in the placebo group and 260 (42.4%) in the UDCA group. Some of those participants had multiple follow-up colonoscopies. Of the participants with multiple follow-up colonoscopy (N = 327), 297 (90.8%) had at least one follow-up colonoscopy no earlier than six months before the three-year anniversary date after randomization and 138 (42.2%) had at least one newly discovered adenoma at their first follow-up colonoscopy. A participant could have newly discovered adenomas at the first colonoscopy and no newly discovered adenomas at the second colonoscopy. This is because at each colonoscopy the participant's colorectal polyps were removed and tested to see if any of them is adenomatous. Instead of fixing the end of the trial exactly at three years, for each participant the actual time of the colonoscopy is used to define the interval of time to first recurrence. The midpoint imputation method is then conducted on the interval censored observations.

Table 1 explores the covariates associated with time to first recurrence and time to censoring for the placebo and UDCA groups separately by fitting PH models to the midpoint imputed data. According to the table, for the placebo group age, gender, previous polyp history, size (≥ 1 *cm*) of the largest baseline adenoma and multiplicity (number of baseline adenomas) are significantly associated with risk of recurrence and previous polyp history is marginally associated with risk of censoring. This indicates that the magnitude of dependent censoring is weak in the placebo group. A working PH model with the five significant covariates as covariates is fitted to the midpoint imputed recurrence time data to derive a risk score of recurrence. The risk score of recurrence is categorized into four groups by the quartiles and the dichotomous previous polyp history is directly used to represent the risk score of censoring. The two categorized risk scores are then used to define risk groups for performing the WKM estimation. We study three WKM methods, WKM^*r*+*c*^(4, 2), WKM^*r*^(4) and WKM^*c*^(2).

**Table 1:** Univariate analysis based on a PH model for the UDCA study.

Time to Recurrence						
	**Placebo**			**UDCA**		
**Variable**	**Hazard Ratio**	**95% CI**	**p-value**	**Hazard Ratio**	**95% CI**	**p-value**

Age	1.026	(1.010,1.042)	<0.01	1.010	(0.995,1.025)	0.20
BMI(≥ 25)	1.210	(0.914,1.602)	0.18	1.464	(1.087,1.970)	0.01
Male	1.312	(1.002,1.719)	0.05	1.235	(0.936,1.630)	0.14
Previous Polyp History	1.378	(1.070,1.774)	0.01	1.029	(0.798,1.327)	0.82
Family History of CRC^*a*^	1.001	(0.766,1.310)	0.99	1.024	(0.774,1.355)	0.87
Size (≥ 1 cm)	1.418	(1.108,1.815)	0.01	1.718	(1.340,2.203)	<0.01
Multiplicity	1.678	(1.311,2.149)	<0.01	1.921	(1.498,2.463)	<0.01

**Time to Censoring**						

Age	1.008	(0.995,1.020)	0.23	1.004	(0.992,1.016)	0.49
BMI(≥ 25)	1.207	(0.949,1.535)	0.13	1.127	(0.899,1.413)	0.30
Male	1.002	(0.799,1.256)	0.99	0.999	(0.802,1.244)	0.99
Previous Polyp History	0.820	(0.650,1.035)	0.09	0.849	(0.687,1.051)	0.13
Family History of CRC	0.944	(0.742,1.199)	0.63	0.777	(0.610,0.989)	0.04
Size (≥ 1 cm)	0.999	(0.798,1.252)	1.00	1.303	(1.051,1.615)	0.02
Multiplicity	0.960	(0.756,1.220)	0.74	1.059	(0.850,1.319)	0.61

^*a*^colorectal cancer.

For the UDCA group, BMI (≥ 25), size (≥ 1 *cm*) of the largest baseline adenoma and multiplicity (number of baseline adenomas) are significantly associated with risk of recurrence and family history of colorectal cancer and size are significantly associated with risk of censoring in the UDCA group. Size is significantly associated with both risk of recurrence and risk of censoring. This indicates that there is potential dependent censoring in the UDCA group. A working PH model with the three significant covariates as covariates is fitted to the midpoint imputed recurrence time data to derive a risk score of recurrence. A working PH model with the two significant covariates as covariates is fitted to the observed censoring time data to derive a risk score of censoring. The two risk scores are standardized to perform principal component analysis to derive two orthogonal components. These two components are categorized separately into 4*2 groups based on their percentiles, where 4 is the number of categories for the first component and 2 is the number of categories for the second component, to define risk groups for performing the WKM estimation. We study three WKM methods, WKM^*r*+*c*^(4, 2), WKM^*r*^(4) and WKM^*c*^(2). In this paper we are interested in estimating the recurrence rate at three years for both the placebo and UDCA groups and the associated odds ratio based on the UDCA study protocol. In addition to the WKM methods, we also calculate the sample proportion of recurrence (Logit), WLogit (with an exponential weight function truncated at three years^1^) and NPMLE.

The results are provided in Table 2. Logit produces a lower recurrence rate compared to the NPMLE and WKM methods for both placebo and UDCA groups. This supports our previous findings [1]. WLogit produces a higher recurrence rate compared to the other methods (Logit, NPMLE and WKM) for both placebo and UDCA groups. All three WKM methods produce a slightly higher recurrence rate for the placebo group and a similar recurrence rate for the UDCA group compared to the NPMLE method, especially for the WKM methods that incorporate risk scores from censoring time into the analysis to adjust for potential dependent censoring. This results in a lower odds ratio 0.734 with a 95% confidence interval (CI) of (0.566, 0.981) and 0.738 with a 95% CI of (0.554, 0.977) for the WKM^*r*+*c*^(4,2) and WKM^*c*^(2), respectively. The 95% CI for both WKM^*c*^(2) and WKM^*r*+*c*^(4,2) does not cover one and indicates that UDCA is associated with a lower risk of recurrence in contrast to the results from the NPMLE, Logit and WLogit methods. The WKM^*r*^(4) method, which only incorporates risk scores from recurrence time into the analysis, produces an odds ratio 0.758 with a 95% CI of (0.572,1.018), which covers one. In summary, the WKM method could provide an adjustment for dependent censoring through using information from the auxiliary variables.

**Table 2:** Estimation of recurrence rate for placebo and UDCA groups at three years.

	Placebo		UDCA		
Method	estimate	standard error	estimate	standard error	Odds Ratio (95% CI)
NPMLE	0.467	0.031	0.414	0.032	0.807 (0.545,1.136)
Logit	0.439	0.020	0.409	0.020	0.887 (0.705,1.122)
WLogit	0.539	0.023	0.504	0.023	0.869 (0.674,1.157)
WKM^*r*+*c *^(4, 2)	0.495	0.027	0.419	0.022	0.734 (0.566,0.981)
WKM^*r *^(4)	0.489	0.027	0.421	0.023	0.758 (0.572,1.018)
WKM^*c *^(2)	0.494	0.028	0.418	0.021	0.738 (0.554,0.977)

### Simulation study

We perform a simulation study to investigate the small sample size properties of the WKM methods under situations with auxiliary variables and independent or dependent censoring. We mainly focus on comparing the estimate of recurrence rate at the end of the trial (three years) between WKM^*c*^, WKM^*r*^, WKM^*r*+*c*^, KM, NPMLE, Logit (sample proportion of recurrence), and WLogit (with an exponential weight function truncated at three years) methods. In addition, we are also interested in exploring the effects of rates of having early colonoscopy and censoring before the end of the trial on the WKM methods.

For each of 500 independent simulated data sets, there are five hypothetical auxiliary variables (*Z*_1_,..., *Z*_5_) independently generated from a *Uniform*(0, 1) distribution. Each participant's recurrence time (*X*) is generated from a hypothetical PH model conditional on the five auxiliary variables, where the hazard function is *λ*_*r*_(*x*) = *t*^0.5 ^* *exp*(-2.0*Z*_1 _+ 0.5*Z*_2 _-2.0*Z*_3 _+ 1.5*Z*_4 _+ 0.5*Z*_5_). For simplicity, we assume every participant only has one follow-up colonoscopy. The follow-up colonoscopy time is generated from a hypothetical distribution truncated at three years (i.e. the end of the trial), in which the parameters of the distribution are selected to control the probability of having early colonoscopy. In a situation with independent censoring, time to early colonoscopy (*T*) is generated from an exponential distribution with a constant hazard *λ *and truncated at three years. In a situation with dependent censoring, time to early colonoscopy is generated from a hypothetical PH model conditional on auxiliary variables with a hazard function *λ*_*e*_(*t*; *Z*_1_,..., *Z*_5_) and truncated at three years. For a participant who has the follow-up colonoscopy at three years, the recurrence time is then either observed and censored in the interval of [0, 3] or right censored at three years. For a participant who has the follow-up colonoscopy before three years, the recurrence time is then either observed and censored in the interval of (0, *T*] or right censored at *T*. In order to perform the KM and WKM methods, each participant is considered either right censored at *T *or three years or having recurrence at the midpoint of the interval (0, *T*] (i.e. *T*/2) or (0, 3] (i.e. 1.5). A working PH model with the five hypothetical auxiliary variables as the covariates is fitted to the midpoint imputed recurrence time data and the observed censoring time data, respectively, to derive two risk scores. Four risk groups are then defined using both risk scores or one of the two risk scores to perform the WKM estimation. A sample size of 200 and various rates of having early colonoscopy, *p*_*ec*_, are considered in this paper. The standard error of each of the 500 datasets for NPMLE is derived from 500 bootstrap samples. The results are provided in Tables 3 and 4. In a situation with independent censoring (Table 3), as expected, the Logit method has the largest bias in estimating the recurrence rate at the end of trial (three years) compared to the other methods in all situations. The bias results in a low coverage rate for the Logit method. The NPMLE, KM, WLogit and WKM methods all produce a point estimate comparable to the true recurrence rate and a coverage rate close to the nominal level. As the rate of having early colonoscopy increases, the bias increases for the KM, NPMLE and WKM methods. The bias results in a coverage rate slightly off from the nominal level for the WKM^*r *^and WKM^*r*+*c *^methods when the rate of having early colonoscopy is approximate 50%. As the number of risk groups increases to 8, the WKM methods produce similar results (not shown here). Of the three WKM methods, WKM^*c *^has the lowest bias and the closest coverage rate to the nominal level. This is because the working PH model used to derive a risk score to summarize risk of recurrence based on the midpoint imputed data can be considered as misspecified. The WKM^*c*^method incorporating information from the auxiliary variables into estimation gains efficiency ranging from 17% to 27% based on the empirical variance, i.e. square of the empirical standard deviation (SD), compared to the NPMLE method and ranging from 4% to 10% compared to the WLogit method. The WKM method gains efficiency by recovering information for interval-censored participants who have early colonoscopy using the auxiliary variables. For the KM method, midpoint imputation is used to impute recurrence time for all interval-censored participants who have early colonoscopy. After imputation, there is no interval-censored observations among those who have early colonoscopy. Hence, the WKM method has a similar efficiency as the KM method.

**Table 3:** Monte Carlo Results: Estimation of recurrence of adenomas at three years (true recurrence rate: 0.495) under a situation of independent censoring with five auxiliary variables.

Method	**Est**^*a*^	Bias	**SD**^*b*^	**SE**^*c*^	**CR**^*d*^
*λ*^*e *^= 0.12; = 0.30; early censoring rate^*g *^= 23.0%; |*r*^*h*^| = 0.35					

NPMLE	0.501	0.006	0.0405	0.0404	93.8
KM	0.485	-0.010	0.0371	0.0381	94.2
Logit	0.417	-0.078	0.0339	0.0348	36.6
WLogit	0.490	-0.005	0.0375	0.0391	96.2
WKM^*r*+*c*^(4,1)	0.482	-0.013	0.0369	0.0375	94.4
WKM^*r*^(4)	0.480	-0.015	0.0366	0.0372	94.4
WKM^*c*^(4)	0.484	-0.011	0.0368	0.0380	94.6

*λ *= 0.17; *p*_*ec *_= 0.40; early censoring rate = 30.5%; |*r*| = 0.36					

NPMLE	0.501	0.006	0.0429	0.0431	94.0
KM	0.481	-0.014	0.0383	0.0394	93.6
Logit	0.390	-0.105	0.0337	0.0344	13.0
WLogit	0.489	-0.006	0.0392	0.0407	94.0
WKM^*r*+*c*^(4, 1)	0.477	-0.018	0.0380	0.0385	92.6
WKM^*r*^(4)	0.474	-0.021	0.0377	0.0380	91.8
WKM^*c*^(4)	0.480	-0.015	0.0376	0.0392	94.0

*λ *= 0.23; *p*_*ec *_= 0.50; early censoring rate = 38.3%; |*r*| = 0.37					

NPMLE	0.506	0.011	0.0460	0.0465	94.8
KM	0.480	-0.015	0.0403	0.0409	94.6
Logit	0.363	-0.132	0.0340	0.0339	2.4
WLogit	0.491	-0.004	0.0414	0.0426	95.6
WKM^*r*+*c*^(4,1)	0.474	-0.021	0.0395	0.0396	90.6
WKM^*r*^(4)	0.471	-0.024	0.0387	0.0391	89.8
WKM^*c*^(4)	0.478	-0.017	0.0392	0.0406	94.0

^*a*^average of 500 estimated recurrence rates.

^*b*^empirical standard deviation of 500 point estimates.

^*c*^average of 500 estimated standard errors.

^*d*^fraction of 95% confidence intervals which contain the true value.

^*e*^hazard rate for time to colonoscopy.

^*f*^proportion of participants with early colonoscopy.

^*g*^right censoring occurs before the end of the trial (3 years).

^*h*^correlation coefficient between the two risk scores.

**Table 4:** Monte Carlo Results: Estimation of recurrence of adenomas at three years (true recurrence rate: 0.495) under a situation of dependent censoring with five auxiliary variables.

Method	Est	Bias	SD	SE	CR
*λ*_*e*_(*t*)^*a *^= *t*^0.1 ^* *exp*(-2.5*Z*_1 _- 0.5*Z*_2 _- 2.0*Z*_3 _- 0.25*Z*_4 _+ 0.5*Z*_5_) *p*_*ec *_= 0.30; early censoring rate = 19.9%; |*r*| = 0.83					

NPMLE	0.447	-0.048	0.0385	0.0389	74.6
KM	0.456	-0.039	0.0376	0.0378	82.2
Logit	0.398	-0.097	0.0347	0.0345	19.0
WLogit	0.460	-0.035	0.0379	0.0384	85.6
WKM^*r*+*c*^(4,1)	0.483	-0.012	0.0368	0.0373	94.0
WKM^*r*^(4)	0.480	-0.015	0.0371	0.0371	92.8
WKM^*c*^(4)	0.479	-0.016	0.0376	0.0376	93.0

*λ*_*e*_(*t*) = *t*^0.1 ^* *exp*(-2.5*Z*_1 _+ 0.4*Z*_2 _- 2.0*Z*_3 _- 0.3*Z*_4 _+ 0.5*Z*_5_) *p*_*ec *_= 0.40; early censoring rate = 27.8%; |*r*| = 0.86					

NPMLE	0.420	-0.075	0.0397	0.0402	54.4
KM	0.434	-0.061	0.0382	0.0388	65.0
Logit	0.356	-0.139	0.0327	0.0338	1.0
WLogit	0.439	-0.056	0.0388	0.0397	72.8
WKM^*r*+*c*^(4,1)	0.471	-0.024	0.0381	0.0385	89.0
WKM^*r*^(4)	0.468	-0.027	0.0384	0.0382	89.6
WKM^*c*^(4)	0.466	-0.029	0.0385	0.0390	87.6

*λ*_*e*_(*t*) = *t*^0.1 ^* *exp*(-2.5 *Z*_1 _+ 0.9*Z*_2 _- 1.5*Z*_3 _- 0.5*Z*_4 _+ 0.5*Z*_5_) *p*_*ec *_= 0.50; early censoring rate = 36.6%; |*r*| = 0.78					

NPMLE	0.407	-0.088	0.0409	0.0428	46.6
KM	0.421	-0.074	0.0395	0.0403	57.2
Logit	0.321	-0.174	0.0321	0.0329	0.0
WLogit	0.429	-0.066	0.0399	0.0415	63.8
WKM^*r*+*c*^(4,1)	0.468	-0.027	0.0408	0.0405	89.0
WKM^*r*^(4)	0.462	-0.033	0.0408	0.0396	86.4
WKM^*c*^(4)	0.457	-0.038	0.0406	0.0416	84.0

^*a*^hazard function for time to colonoscopy.

In a situation with dependent censoring (Table 4), as expected, all Logit, WLogit, KM, and NPMLE methods produce biased estimates of recurrence rate in all situations, especially for the Logit method. The bias for all four methods increases with the rate of having early colonoscopy. The bias results in low coverage rates for all Logit, WLogit, KM, and NPMLE methods, especially for the Logit method. The bias of NPMLE, KM, and WLogit methods is because these three methods assume that the censoring is independent of risk of recurrence. All three WKM methods produces point estimates comparable to the true recurrence rate and the coverage rates comparable to the nominal level in a situation that the rate of having early colonoscopy is 30%. When the rate of having early colonoscopy increases, we do observe the bias increases for the all three WKM methods, WKM^*c*^, WKM^*r *^and WKM^*r*+*c*^. This results in a slightly off coverage rate compared to the nominal level, especially when the rate of having early colonoscopy is 50%. All three WKM methods produce similar estimates in all situations. This is because the two risk scores are highly correlated. As the number of risk groups increases to 8, the WKM methods produce similar results (not shown here).

In summary, the results in Tables 3 and 4 show that the Logit method tends to produce biased estimates of recurrence rate at the end of the trial in both situations of independent and dependent censoring. The KM, NPMLE and WLogit methods all can produce a reasonable estimate in a situation with independent censoring but are associated with bias in a situation with dependent censoring. The WKM method, which adjusts for the potential dependent censoring using the information from auxiliary variables, can gain efficiency compared to the NPMLE and WLogit methods and reduce bias due to dependent censoring compared to the KM, NPMLE and WLogit methods while estimating recurrence rate at the end of the trial.

## Discussion

Simply using midpoint imputation to handle interval-censored observations highly depends on the lengths of intervals and might produce biased survival estimates and misleading results, especially at early time points. However, in this paper, we focus on estimating the recurrence rate at the end of the trial and have shown that midpoint imputation will not produce a biased estimate of recurrence rate at the end of the trial under an assumption of independent censoring. In simulation studies we do not observe significant bias associated with the WKM method in a situation with independent censoring. Only in a situation with dependent censoring and a higher rate of having early colonoscopy, do we observe a greater bias associated with the WKM method. This is mainly because the working PH model used to derive a risk score to summarize risk of recurrence is based on the midpoint imputed data, which in fact are not the true observed recurrence time data, and can be considered as misspecified and the two continuous risk scores are categorized to define risk groups. Hence, there are still some remnants of dependent censoring. The standard error of the survival estimator derived from the midpoint imputed data could be underestimated since midpoint imputation assumes that the recurrence times are exactly known but in fact the exact recurrence times are not observed [19]. In simulation studies, we do not observe underestimation of the standard error associated with the WKM methods in both situations of independent and dependent censoring. This is probably because in a colorectal polyp prevention trial often over 50% of participants had their only follow-up colonoscopy at the end of the trial. Those participants were either interval censored or right censored at the end of the study. The information they provide towards estimation of recurrence rate at the end of the study simply reduces to a binary outcome and their follow-up lengths provide little information with regard to the actual recurrence time. We suspect this might stabilize the tail problem because we do not observe unstable estimates in the simulation study.

In a situation with multiple auxiliary variables, we fit two working PH models to the midpoint imputed data to reduce multiple auxiliary variables into two scalars. The models are only used as a convenience in calculating the risk scores to create a categorical variable, which is predictive of risk of recurrence or censoring, to implement the weighted KM method. More sophisticated and computationally intensive approaches for fitting the working model could be used, such as a proportional hazard model for interval-censored data, but we suspect that would not lead to a significant reduction in the bias, which is the major concern under a situation with dependent censoring for the WKM method. In addition, parametric assumptions connected with the statistical model are only employed to define the risk scores. As a result, the reliance on the PH model is weaker for the WKM approach. However, the performance of the WKM method using predictive covariates of recurrence to improve efficiency in estimation of the recurrence rate in a dependent censoring situation will depend on the strength of the association between these auxiliary variables and recurrence.

## Conclusion

The research in this paper uses midpoint imputation to handle interval-censored observations and then uses the information from auxiliary variables to adjust for dependent censoring by incorporating them into the weighted Kaplan-Meier estimation. This approach handles a situation with multiple auxiliary variables by deriving two risk scores from two working PH models. Although the idea of this approach might appear simple, the simulation results do show that the weighted Kaplan-Meier approach can gain efficiency and reduce bias due to dependent censoring. In contrast, the sample proportion of recurrence (Logit) tends to underestimate the recurrence rate and the KM, NPMLE and WLogit methods, which all rely heavily on the assumption of independent censoring, could produce biased estimates in a situation with dependent censoring. Hence, the method that does not account for variable follow-up lengths or dependent censoring while estimating the recurrence rate at the end of the trial could produce misleading conclusions as indicated in the data analysis section.

## Competing interests

The authors declare that they have no competing interests.

## Authors' contributions

CHH conceived of the study and conducted simulation with inputs from QL and DSA. CHH wrote the first draft of the manuscript. Subsequent drafts were edited by CHH, QL and DSA to ensure the content accuracy. All authors reviewed and approved the final version of the manuscript.

## Pre-publication history

The pre-publication history for this paper can be accessed here:

http://www.biomedcentral.com/1471-2288/9/66/prepub
